# Long non-coding RNA OIP5-AS1 suppresses multiple myeloma progression by sponging miR-27a-3p to activate TSC1 expression

**DOI:** 10.1186/s12935-020-01234-7

**Published:** 2020-05-07

**Authors:** Yong Wang, Haibao Wang, Jianwei Ruan, Wenbiao Zheng, Zeyu Yang, Weiwei Pan

**Affiliations:** Department of Orthopaedic, Taizhou Municipal Hospital, No. 381, Zhongshan East Road, Jiaojiang District, Taizhou, 318000 Zhejiang China

**Keywords:** Multiple myeloma, lncRNA OIP5-AS1, miR-27a-3p, TSC1

## Abstract

**Background:**

Multiple myeloma (MM) is a prevalent hematological malignancy. Long noncoding RNAs are correlated with the development of MM. In this project, the function of lncRNA opa interacting protein 5-antisense 1 (OIP5-AS1) in MM and the potential mechanistic pathway were explored.

**Methods:**

The expression of OIP5-AS1, microRNA (miR)-27a-3p and tuberous sclerosis 1 (TSC1) was analyzed by quantitative real-time polymerase chain reaction (qRT-PCR) assay. Cell proliferation was assessed by Cell Counting Kit-8 (CCK-8) assay, colony formation assay and Bromodeoxyuridine (BrdU) staining. And cell apoptosis was evaluated by flow cytometry assay. Cell metastasis was assessed utilizing transwell assay. Western blot analysis was employed to detect protein level. The target relation between miR-27a-3p and OIP5-AS1 or TSC1 was confirmed via dual-luciferase reporter assay and RNA immunoprecipitation assay. Tumor xenograft assay was conducted to measure the function of OIP5-AS1 in vivo.

**Results:**

The expression levels of OIP5-AS1 and TSC1 were decreased in MM, whereas miR-27a-3p was upregulated. High level of OIP5-AS1 could predict favourable prognosis of MM patients. Overexpression of OIP5-AS1 inhibited cell viability, colony formation ability, migration and invasion, induced cell cycle arrest in G1 phase and apoptosis of MM cells in vitro as well as repressed tumorigenesis in vivo. MiR-27a-3p was a target of OIP5-AS1, and reversed the impact of OIP5-AS1 on MM cells. MiR-27a-3p directly targeted TSC1. Silencing of miR-27a-3p repressed MM progression by elevating TSC1 expression. OIP5-AS1 upregulated TSC1 by sponging miR-27a-3p.

**Conclusion:**

OIP5-AS1 repressed multiple myeloma progression by regulating miR-27a-3p/TSC1 axis.

## Background

Multiple myeloma (MM) ranks as the second most prevalent hematological cancer, and often harms the aged population [1]. MM makes up of approximately 20% of deaths associated with malignancies, with diverse clinical manifestations, such as bone malformation, hypercalcemia, anemia, renal impairment, recurrent infection and so on [2]. Although current therapeutic approaches have improved the outcomes of patients with MM [3], better management of multiple myeloma is still urgently needed.

Long noncoding RNAs (lncRNAs) are 200 nucleotides to 1000 kilobases long, without protein-coding ability due to the absence of open reading frames [4]. Former publication presents that lncRNAs harbor potential to be biomarkers and/or treatment targets of human malignancies, and involve in numerous biological processes during tumorigenesis, including MM [5, 6]. For example, the obvious upregulation of lncRNA taurine-upregulated gene 1 (TUG1) was discovered in the serum of MM patients, and might be an independently predictive biomarker for MM diagnosis [7]. High expression of lncRNA small nucleolar RNA host gene 18 (SNHG18) and semaphorin 5A (SEMA5A) could indicate unfavorable prognosis of patients with MM [8]. In addition, lncRNA XLOC_013703 modulated survival and proliferation of MM cells through nuclear factor-κB pathway, functioning as a potential therapy target for MM [9]. LncRNA opa interacting protein 5-antisense 1 (OIP5-AS1) could facilitate complicated cellular mechanisms in the development of human malignant tumors [10]. Previous literature demonstrated that OIP5-AS1 was dysregulated in MM, and took part in regulating MM cell proliferation and apoptosis [11]. Here, we aimed to explore novel mechanistic pathway of OIP5-AS1 in the cellular processes of MM.

MicroRNAs (miRNAs) are a category of well-studied noncoding RNAs, consisting of about 22 nucleotides, and affect the stability and translation of mRNAs [12]. Many dysregulated miRNAs are implicated with MM development, and can act as prognostic bioindicators and therapy targets [13]. MiR-27a-3p is mature isoform of miR-27a, which participating in the regulation on certain signaling pathways in many cancers, like AKT signaling pathway, Wnt/β-catenin signaling pathway, Ras/MEK/ERK signaling pathway, TGF-β signaling pathway [14]. MiR-27a exerts diverse functions in human cancer tumorigenesis, proliferation and apoptosis, invasion and migration, angiogenesis by directly binding to the 3′ untranslated region (3′UTR) of target messenger RNA (mRNA) [15]. MiR-27a was reported to have biological role in MM by targeting Sprouty homolog 2 (SPRY2) [16]. Whether miR-27a-3p partaking in OIP5-AS1-modulated MM progression is unknown.

Tuberous sclerosis 1 (TSC1) was involved in the regulation of PI3K/Akt/mTOR signaling pathway in MM by serving as a target of miR-451 [17]. Here, we intended to explore the effect of TSC1 on OIP5-AS1-mediated MM progression.

In the current project, we examined the expression pattern and the functional effects of lncRNA OIP5-AS1 on the cellular behaviors of multiple myeloma NCI-H929 and MM1.S cells in vitro and on the tumor growth in vivo, and explored the potential mechanism.

## Materials and methods

### Clinical samples

The current research was ratified by the Ethics Committee of Taizhou Municipal Hospital. Bone marrows were obtained from 38 patients with MM and 25 healthy hematopoietic stem cell donors enrolled at Taizhou Municipal Hospital. 38 MM patients were recently diagnosed based on 2014 International Myeloma Working Group (IMWG) updated criteria for the diagnosis of multiple myeloma, and the clinical details of these 38 patients were recorded. Normal bone marrows from 25 donors worked as control (Normal). All participators signed written informed consents.

### Cell culture and transfection

Human MM NCI-H929 (CRL-9068), MM1.S (CRL-2974) and RPMI-8226 (CRM-CCL-155) cells were purchased from American Type Culture Collection (ATCC; Manassas, VA, USA) and maintained in Roswell Park Memorial Institute 1640 Medium (RPMI1640; Gibco, Grand Island, NY, USA) mixed with 10% fetal bovine serum (FBS, Gibco), 100 U/mL penicillin and 100 mg/mL streptomycin in a 5% CO_2_ humidified incubator at 37 °C.

The overexpression vector of OIP5-AS1 (LncRNA OIP5-AS1) and its negative control (Lnc-NC) were synthesized by Hanbio Biotechnology Co., ltd (Shanghai, China). The small interference RNA (siRNA) against OIP5-AS1 (si-LncRNA OIP5-AS1) and its negative control (si-NC), siRNA against TSC1 (si-TSC1) and its negative control (scramble) were generated via Genechem (Shanghai, China). MiR-27a-3p mimic (miR-27a-3p) and its negative control (NC), miR-27a-3p inhibitor (anti-miR-27a-3p) and its negative control (anti-NC) were provided by GenePharma Co. Ltd. (Shanghai, China). The above oligonucleotides (40 mM) or plasmids (2 μg) were transfected into MM cells with the aid of Lipofectamine 3000 (Life Technologies Corporation, Carlsbad, CA, USA). 48 h later, cells were harvested for subsequent assays.

### Quantitative real-time polymerase chain reaction (qRT-PCR)

To isolate total RNA from bone marrows or MM NCI-H929, MM1.S and RPMI-8226 cells, TRIzol Reagent (Beyotime, Shanghai, China) was used. Then 1 μg RNA served as template for cDNA synthesis utilizing BeyoRT™ III M-MLV reverse transcriptase (Beyotime) or miRNA First-Strand Synthesis Kit (Clontech, Mountain View, CA, USA). QRT-PCR was performed with diluted cDNA and GoScript Reverse Transcription System (Promega, Madison, WI, USA) or miRNA qRT-PCR TB Green Kit (Clontech) on StepOnePlus™ Real-time PCR Systems (Applied Biosystems, Foster City, CA, USA). The relative expression of genes was normalized to Glyceraldehyde-3-phosphate dehydrogenase (GAPDH, for OIP5-AS1 and TSC1) or U6 (for miR-27a-3p) exploiting 2^−ΔΔCt^ method. All primers were acquired from Sangon (Shanghai, China): OIP5-AS1 (Forward, 5′-TGCGAAGATGGCGGAGTAAG-3′; Reverse, 5′-CCACCACCTAAGCACCTGTT-3′); TSC1 (Forward, 5′-CAACAGGCGTCTTGGTGTTG-3′; Reverse, 5′-ACACACTGGCATGGAGATGG-3′); GAPDH (Forward, 5′-CACCATCTTCCAGGACGAG-3′; Reverse, 5′-CCTTCTCCATGGTGGTGAAGA-3′); miR-27a-3p (Forward, 5′-TGCGGTTCACAGTGGCTAAG-3′; Reverse, 5′-CTCAACTGGTGTCGTGGA-3′); U6 (Forward, 5′-CGCTAGCACATATCGGCTA-3′; Reverse, 5′-TTCTGCGACGAATTTGTCAT-3′).

### Cell viability assay

Cell Counting Kit-8 (CCK-8, Beyotime) reagent was applied to monitor the viability of MM NCI-H929, MM1.S and RPMI-8226 cells referring to the protocols supplied by the manufacturer. Cells were inoculated in 96-well plate with a density of 5 × 10^3^ cells per well and transfected for 24 h, 48 h or 72 h. After 10 μL CCK-8 reagent was introduced, cells were maintained for additional 1 h at 37 °C. The absorbance of each well at 450 nm was recorded using a Microplate Reader (Bio-Rad, Hercules, CA, USA).

### Colony formation assay

NCI-H929, MM1.S and RPMI-8226 cells were seeded in 6-well plate (300 cells/well). After regular culture for 2 weeks, formed colonies (recognized as > 50 cells/colony) were fixed with methanol and stained with crystal violet, then counted under a microscope. The colony formation rate was counted using the formula: number of cells generating colonies/number of the seeded cells.

### Bromodeoxyuridine (BrdU) staining

This assay was also employed to monitor cell proliferation using BrdU Cell Proliferation ELISA Kit (ab126556; Abcam, Shanghai, China) based on the user’s manual. At 24 h, 48 h or 72 h post transfection, NCI-H929, MM1.S and RPMI-8226 cells (~ 1 × 10^4^) in 100 μL Medium were seeded into 96-well plates and maintained for 24 h. Then, cells were disposed with 20 μL diluted BrdU reagent for 6 h, fixed with Fixing solution and incubate for 30 min, and recognized with anti-BrdU antibody and peroxidase Goat Anti-Mouse IgG Conjugate. After washing and substrate reaction, the absorbance of each well at 450 nm was measured.

### Flow cytometry assay for cell apoptosis

Annexin V-fluoresceine isothiocyanate (FITC) Apoptosis Detection Kit (Beyotime) was exploited to detect the apoptotic rate of NCI-H929, MM1.S and RPMI-8226 cells. 2 × 10^5^ cells seeded in 6-well plate were incubated for 24 h. Later, 10 μL Annexin V-FITC and propidine iodide (PI) solution were added. 15 min later, apoptotic cells were measured by a flow cytometer (Beckman Coulter, Fullerton, CA, USA) and analyzed using FACScan. The apoptotic rate was computed by the analysis of the proportion of cells at Annexin V +/PI ± .

### Flow cytometry assay for cell cycle

After transfection, NCI-H929, MM1.S and RPMI-8226 cells were harvested, washed by pre-cold phosphate buffer saline (PBS) and fixed at 4 °C overnight. Later, cells were stained with PI away from light for 15 min. The distribution of cells at G1 phase, S phase, and G2 phase was monitored using flow cytometer.

### Transwell assay

The invasion and migration capacities of NCI-H929, MM1.S and RPMI-8226 cells were evaluated by transwell assay with Transwell chambers (Corning Inc., Corning, NY, USA). For invasion ability detection, 4 × 10^4^ cells in serum-free medium were inoculated into the upper chambers coated with Matrigel (Corning Inc.). For migration ability detection, 1 × 10^4^ cells in serum-free medium were seeded into upper chambers. The lower ones were filled with the medium embracing 10% FBS. At 24 h post incubation, migrated or invasive cells were dyed with 0.1% crystal violet and counted under a light microscope (×200 magnification).

### Western blot

At first, bone marrows or MM NCI-H929, MM1.S and RPMI-8226 cells were subjected for protein isolation utilizing protein extraction kit (Solarbio, Beijing, China). After quantified with bicinchoninic acid protein assay kit (Beyotime), 30 μg protein samples were loaded on 12% sodium dodecyl sulfate–polyacrylamide gel, then transferred onto polyvinylidene difluoride membranes (Pall Corporation, East Hills, NY, USA). The membranes were blocked in skim milk, followed by incubation with specific primary antibodies anti-Caspase 3 antibody (ab184787; Abcam, 1:1000 dilution), anti-Cleaved caspase-1 (#89332S; Cell Signaling Technology, Danvers, MA, USA, 1:1500 dilution), anti-γ-H2AX (ab195190; Abcam, 1:1000 dilution), anti-Cyclin D1 antibody (ab226977; Abcam, 1:2000 dilution), anti-p21 antibody (ab109520; Abcam, 1:3000 dilution), anti-Ki-67 (ab92742; Abcam, 1:2000 dilution), anti-matrix metalloproteinase 9 (anti-MMP9, ab38898; Abcam, 1:2000 dilution), anti-MMP7 (ab205525; Abcam, 1:2000 dilution), anti-MMP10, (ab38930; Abcam, 1:1500 dilution), anti-TSC1 antibody (ab200728; Abcam, 1:2000 dilution), anti-Proliferating Cell Nuclear Antigen (anti-PCNA, ab92552; Abcam, 1:2000 dilution) or anti-GAPDH antibody (ab181602; Abcam, 1:5000 dilution) as loading control, then probed in secondary antibody conjugated with horseradish peroxidase (ab205718; Abcam, 1:5000 dilution). The protein bands were visualized and analyzed using a chemiluminescence reagent (ECL) kit (Beyotime).

### Dual-luciferase reporter assay

Using the Starbase (http://starbase.sysu.edu.cn/index.php), the binding sites between miR-27a-3p and OIP5-AS1 or TSC1 were searched. The wild-type luciferase reporter vectors (LncRNA OIP5-AS1-wt and TSC1-wt) and mutant-type ones (LncRNA OIP5-AS1-mut and TSC1-mut) were established by inserting corresponding sequences into luciferase reporter vector pGL3 (Promega, Madison, WI, USA). The constructed vectors and miR-27a-3p or NC were cotransfected into NCI-H929 and MM1.S cells. 24 h later, luciferase activity was evaluated exploiting Dual-Lucy Assay Kit (Solarbio).

### RNA immunoprecipitation (RIP) assay

RIP assay was hired to further confirm the target connection between miR-27a-3p and OIP5-AS1 or TSC1 with the EZ-Magna RIP Kit (Millipore, Billerica, MA, USA). In short, NCI-H929 and MM1.S cells were lysed and interacted with antibodies against Ago2 or IgG on the surface of beads. After digestion with Proteinase K, the immunoprecipitated RNA was extracted, purified and utilized for detection for the levels of lncRNA OIP5-AS1, miR-27a-3p and TSC1.

### Xenograft assay

The animal test was endorsed by the Animal Care and Use Committee of Taizhou Municipal Hospital. BALB/c nude mice (4-week-old, male) procured from Shanghai Experimental Animal Center of the Chinese Academy of Sciences (Shanghai, China) were subcutaneously injected with NCI-H929 or MM1.S cells (~ 2 × 10^6^) stably transfected with LncRNA OIP5-AS1 (n = 5) or Lnc-NC (n = 5). The volume of generated tumor (volume = width^2^ × length/2) in mice was recorded once a week. At 5 weeks post injection, the generated tumor was excised, weighed and subjected for qRT-PCR and western blot assays.

### Statistical analysis

Where not specified, each experiment was implemented 3 times. All data were exhibited as mean ± standard deviation. Comparison between two or more groups was executed by two-tail Student’s *t* test or analysis of variance with Tukey post hoc test utilizing GraphPad Prism 6 (GraphPad, La Jolla, CA, USA). The correlation between expression of lncRNA OIP5-AS1 and clinicopathological characteristics of MM patients was evaluated via Chi square test (χ^2^ test). Kaplan–Meier analysis was employed to examine the survival rate of MM patients with low or high expression of OIP5-AS1. Correlation among the expression of OIP5-AS1, miR-27a-3p and TSC1 was analyzed via Pearson correlation analysis. Statistical significance was accepted when *P *< 0.05.

## Results

### LncRNA OIP5-AS1 was low expressed in bone marrows of MM patients

**Fig. 1:**
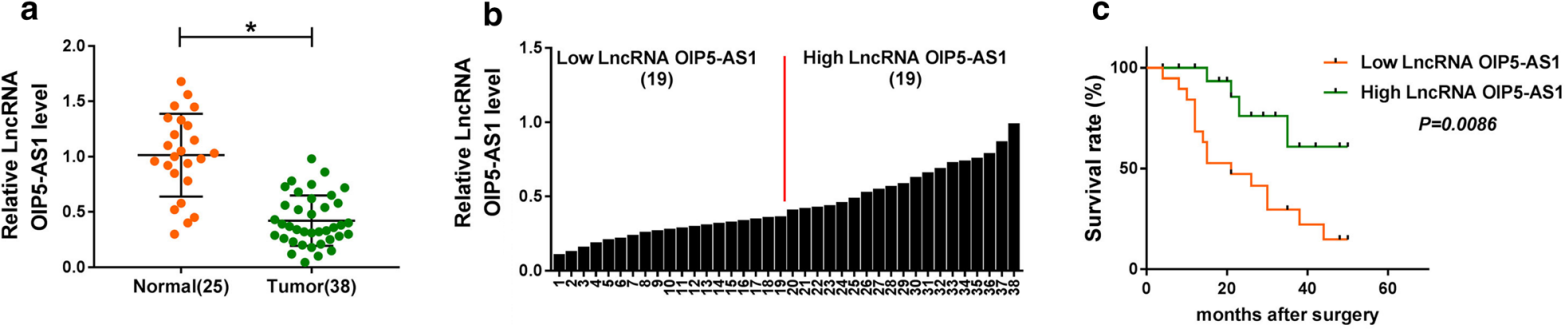
LncRNA OIP5-AS1 was low expressed in bone marrows of MM patients. **a**, **b** The relative expression of lncRNA OIP5-AS1 in bone marrows of 38 MM patients and 25 healthy donors was tested by qRT-PCR assay. **c** Kaplan–Meier analysis for the prognosis of 38 MM patients was done. ^*^P < 0.05

The relative expression of lncRNA OIP5-AS1 was analyzed by qRT-PCR assay. As we can see in Fig. 1a, in contrast with bone marrows of healthy donors, lncRNA OIP5-AS1 was downregulated in the bone marrows of MM patients. The relative expression of lncRNA OIP5-AS1 of 38 bone marrows was displayed in Fig. 1b to indicate the middle expression level of lncRNA OIP5-AS1, so as to divide 38 cases into Low LncRNA OIP5-AS1 group (n = 19) and High LncRNA OIP5-AS1 group (n = 19). Clinicopathological characteristics of these 38 MM patients were displayed in Table 1, and OIP5-AS1 expression was correlated with ISS stage and IMWG risk stratification. Kaplan–Meier analysis revealed that high level of OIP5-AS1 could predict nice prognosis of MM patients (Fig. 1c). Collectively, OIP5-AS1 was downregulated in MM.

**Table 1:** Correlation between the expression of lncRNA OIP5-AS1 in multiple myeloma patients and clinicopathological characteristics

Clinicopathologic feature	n	Expression of lncRNA OIP5-AS1	*P* Value
		mean ± SD	
Age (years)			0.2970
≥ 61	20	0.65 ± 0.06	
< 61	18	0.62 ± 0.11	
Sex			0.6432
Man	18	0.63 ± 0.08	
Woman	20	0.64 ± 0.05	
Type of monoclonal immunoglobulin			0.5162
IgG	18	0.66 ± 0.06	
IgA	12	0.64 ± 0.05	
IgD	3	0.61 ± 0.12	
Light chain only	5	0.63 ± 0.04	
Peripheral blood cell count			
WBC, × 109/L			0.3214
< 5.40	19	0.58 ± 0.08	
≥ 5.40	19	0.67 ± 0.11	
Hb, g/L			
< 83.5	19	0.62 ± 0.07	0.5879
≥ 83.5	19	0.63 ± 0.03	
PLT, × 109/L			
< 127	19	0.64 ± 0.09	0.5213
≥ 127	19	0.61 ± 0.06	
ISS stage			< 0.001***
I	8	1.03 ± 0.08	
II	14	0.71 ± 0.04	
III	16	0.43 ± 0.09	
IMWG risk stratification			< 0.001***
Low risk	7	1.2 ± 0.05	
Intermediate risk	19	0.68 ± 0.12	
High risk	12	0.59 ± 0.08	

SD, standard deviation; WBC, white blood cell; Hb, hemoglobin; PLT, blood platelet; ISS, International Staging System; IMWG, International Myeloma Working Group. ***P < 0.001

### Overexpression of OIP5-AS1 inhibited proliferation and metastasis, but promoted apoptosis of MM cells

**Fig. 2:**
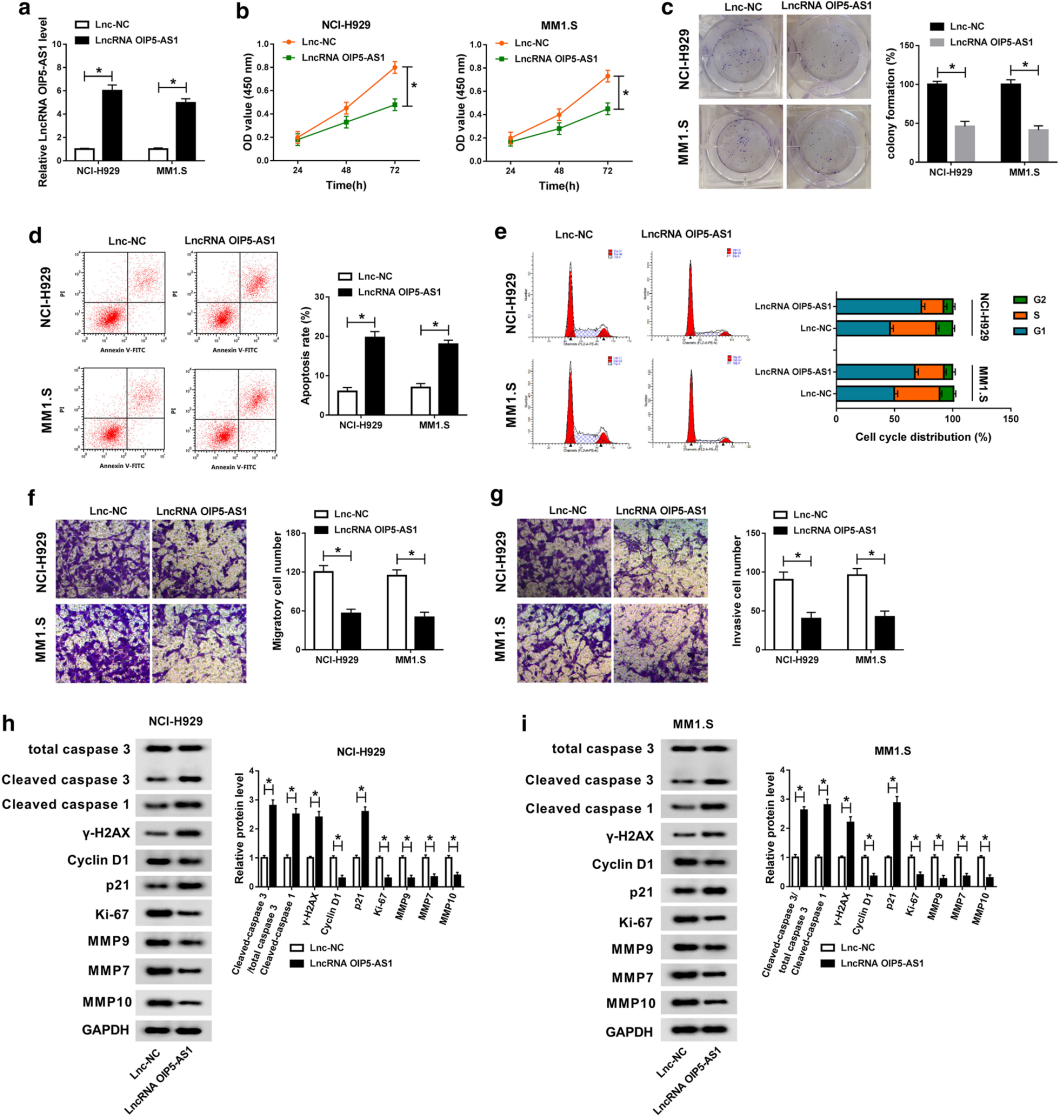
Overexpression of OIP5-AS1 inhibited proliferation and metastasis, but promoted apoptosis of MM cells. MM NCI-H929 and MM1.S cells were transfected with LncRNA OIP5-AS1 or Lnc-NC. **a** The relative expression of lncRNA OIP5-AS1 in transfected cells was analyzed by qRT-PCR assay. **b** Cell viability of transfected cells was analyzed by CCK-8 assay. **c** The colony formation ability of transfected cells was monitored via colony formation assay. **d**, **e** The apoptosis rate and cell cycle distribution were tested through flow cytometry assay. **f**, **g** The migration and invasion abilities of transfected cells were examined by transwell assay. **h**, **i** The protein levels of Cleaved caspase 3/total caspase 3, Cleaved caspase 1, γ-H2AX, Cyclin D1, p21, Ki-67, MMP9, MMP7 and MMP10 were evaluated by western blot analysis. ^*^P < 0.05

To figure out the function of lncRNA OIP5-AS1 in the cellular behaviors of MM in vitro, NCI-H929 and MM1.S cells with OIP5-AS1 overexpression were constructed by transfection, and cells transfected with Lnc-NC served as control. The transfection efficiency was determined by qRT-PCR assay (Fig. 2a). CCK-8 assay uncovered the inhibitory effect of OIP5-AS1 on the viability of NCI-H929 and MM1.S cells (Fig. 2b). As shown in Fig. 2c, upregulation of OIP5-AS1 blocked the colony formation capacity of NCI-H929 and MM1.S cells. Besides, BrdU staining assay showed that enforced expression of OIP5-AS1 decreased the proliferation of MM cells (Additional file 1: Fig. S1A). Flow cytometry assay suggested that overexpression of OIP5-AS1 promoted cell apoptosis and cell cycle arrest at G1 phrase (Fig. 2d, e). Moreover, gain of OIP5-AS1 lowered the number of migrated and invaded NCI-H929 and MM1.S cells (Fig. 2f, g). By performing western blot analysis, we found the increase of Cleaved caspase 3/total caspase 3, Cleaved caspase 1, γ-H2AX and p21 levels, as well as the decrease of Cyclin D1, Ki-67, MMP9, MMP7 and MMP10 levels in NCI-H929 and MM1.S cells transfected with LncRNA OIP5-AS1 (Fig. 2h, i). Likewise, overexpression of OIP5-AS1 triggered the proliferation and metastasis inhibition and apoptosis augment of MM RPMI-8226 cells (Additional file 1: Fig. S2A–I). In sum, introduction of OIP5-AS1 repressed proliferation and metastasis, but facilitated apoptosis of MM NCI-H929 and MM1.S cells.

### LncRNA OIP5-AS1 directly targeted miR-27a-3p

**Fig. 3:**
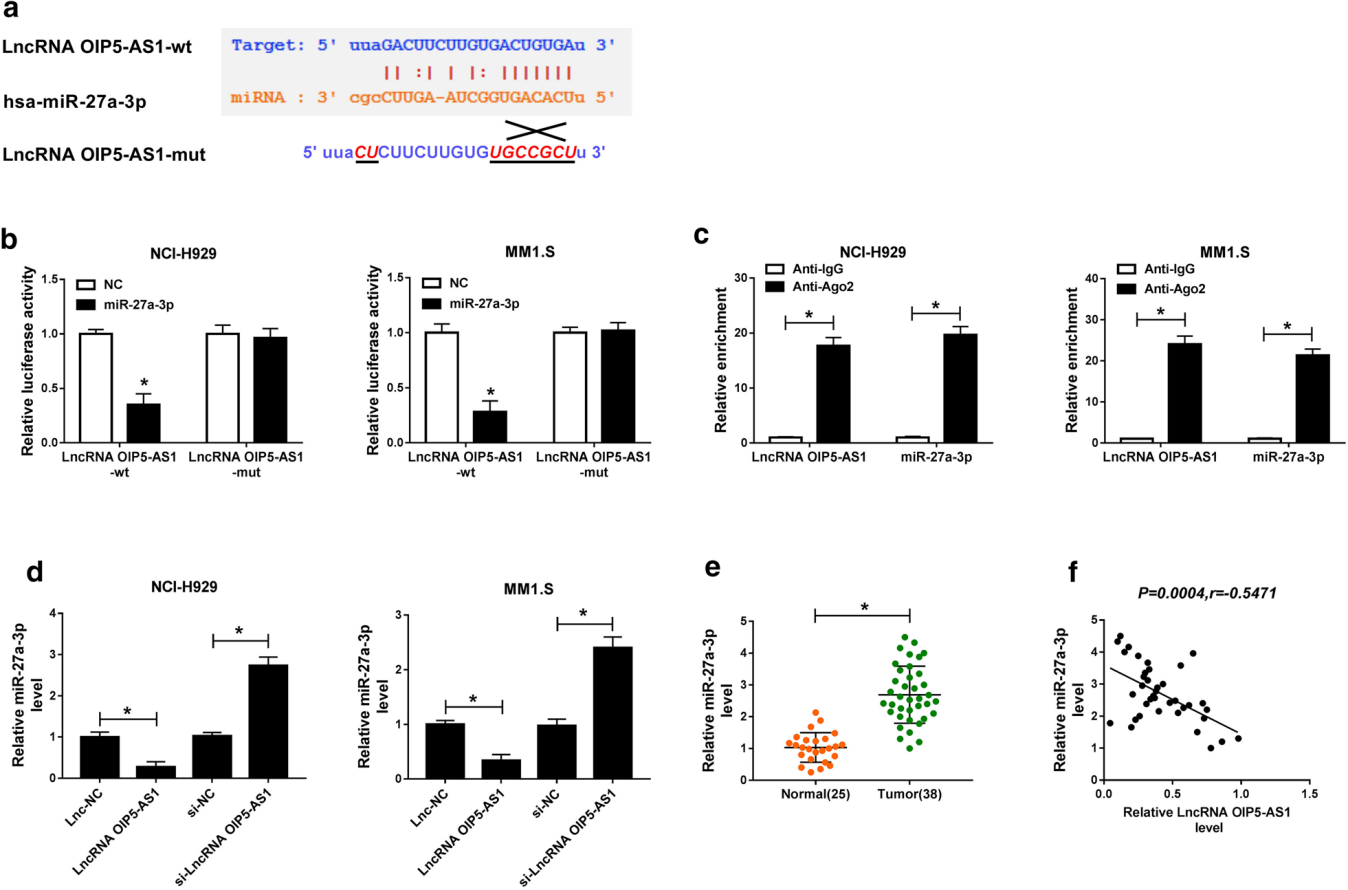
LncRNA OIP5-AS1 directly targeted miR-27a-3p. **a** The binding sites between OIP5-AS1 and miR-27a-3p as well as the mutant were shown. **b** Dual-luciferase reporter assay was carried out to measure the luciferase activity in LncRNA OIP5-AS1-wt group and LncRNA OIP5-AS1-mut group of NCI-H929 and MM1.S cells. **c** The levels of OIP5-AS1 and miR-27a-3p were evaluated after Ago2 or IgG RIP by qRT-PCR assay. **d** The expression of miR-27a-3p in NCI-H929 and MM1.S cells transfected with Lnc-NC, LncRNA OIP5-AS1, si-NC or si-LncRNA OIP5-AS1 was analyzed by qRT-PCR assay. **e** The relative expression of miR-27a-3p in bone marrows of 38 MM patients and 25 healthy donors was tested by qRT-PCR assay. **f** The correlation between the expression of OIP5-AS1 and miR-27a-3p was determined via Pearson correlation analysis. ^*^P < 0.05

Next, we aimed to clarify how OIP5-AS1 affected the development of multiple myeloma. The target miRNAs of OIP5-AS1 were forecasted by Starbase. MiR-27a-3p was found to contain binding sites to OIP5-AS1 (Fig. 3a). The data of dual-luciferase reporter assay showed that miR-27a-3p mimic apparently repressed the luciferase activity in LncRNA OIP5-AS1-wt group of NCI-H929 and MM1.S cells, while it did not alter the luciferase activity in LncRNA OIP5-AS1-mut group (Fig. 3b). RIP assay indicated that both OIP5-AS1 and miR-27a-3p were enriched in the Anti-Ago2 group of NCI-H929 and MM1.S cells (Fig. 3c). Besides, gain of OIP5-AS1 significantly constrained miR-27a-3p expression, while lack of OIP5-AS1 triggered opposite results in NCI-H929 and MM1.S cells (Fig. 3d). Obviously, miR-27a-3p was upregulated in the bone marrows of MM patients versus that in bone marrows of healthy donors (Fig. 3e), and was negatively correlated with OIP5-AS1 expression in bone marrows of 38 MM patients (Fig. 3f). Therefore, miR-27a-3p was a target of OIP5-AS1 and was negatively regulated by OIP5-AS1.

### Upregulation of miR-27a-3p counteracted the effects of OIP5-AS1 on the cellular behaviors of MM cells

**Fig. 4:**
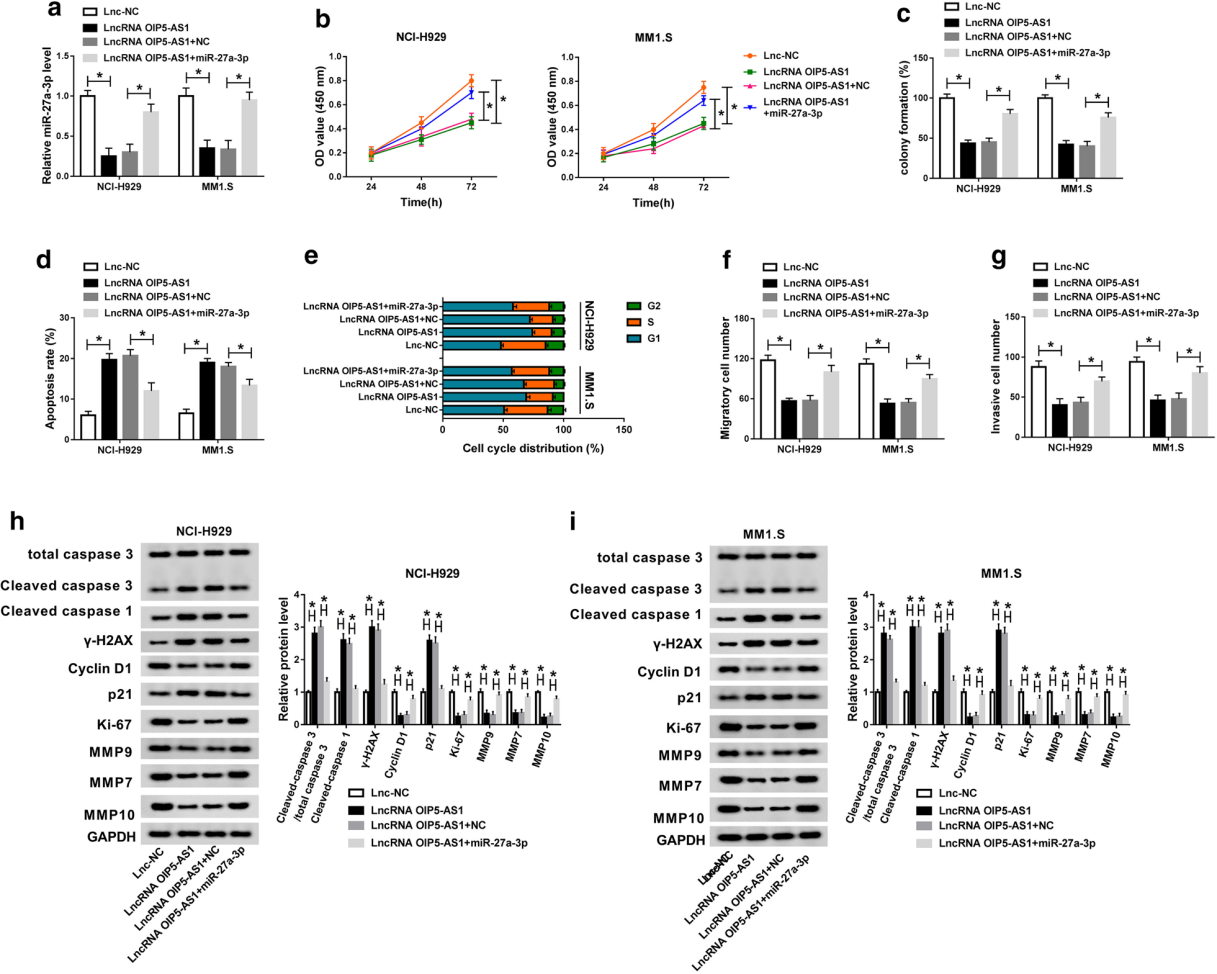
Upregulation of miR-27a-3p counteracted the effects of OIP5-AS1 on the cellular behaviors of MM cells. MM NCI-H929 and MM1.S cells were transfected with Lnc-NC, LncRNA OIP5-AS1, LncRNA OIP5-AS1 + NC or LncRNA OIP5-AS1 + miR-27a-3p. **a** The relative expression of miR-27a-3p in transfected cells was detected by qRT-PCR assay. **b** Cell viability of transfected cells was analyzed by CCK-8 assay. **c** The colony formation ability of transfected cells was measured via colony formation assay. **d**, **e** The apoptosis rate and cell cycle distribution were detected through flow cytometry assay. **f**, **g** The migration and invasion abilities of transfected cells were evaluated by transwell assay. **h**, **i** Protein levels of Cleaved caspase 3/total caspase 3, Cleaved caspase 1, γ-H2AX, Cyclin D1, p21, Ki-67, MMP9, MMP7 and MMP10 were examined by western blot analysis. ^*^P < 0.05

We then performed rescue experiment to explore the way of OIP5-AS1 altering the cellular behaviors of NCI-H929 and MM1.S cells. It was clear that the declined level of miR-27a-3p induced by OIP5-AS1 was recovered by additional miR-27a-3p mimic (Fig. 4a). Furthermore, the inhibitory effects of OIP5-AS1 on the cell viability, colony formation ability and proliferative ability of NCI-H929 and MM1.S cells were relieved due to miR-27a-3p mimic (Fig. 4b, c, Additional file 1: Fig. S1B). Gain of miR-27a-3p also attenuated OIP5-AS1-induced the apoptosis and cell cycle arrest at G1 phrase, which was disclosed by flow cytometry (Fig. 4d, e). What’ more, addition of miR-27a-3p restored the metastasis inhibition caused by OIP5-AS1 (Fig. 4f, g). Besides, overexpression of miR-27a-3p mitigated the influence of OIP5-AS1 on the protein levels of Cleaved caspase 3/total caspase 3, Cleaved caspase 1, γ-H2AX, Cyclin D1, p21, Ki-67, MMP9, MMP7 and MMP10 (Fig. 4h, i). Above results showed that OIP5-AS1 suppressed MM progression by downregulating miR-27a-3p in vitro.

### MiR-27a-3p could target TSC1 in MM

**Fig. 5:**
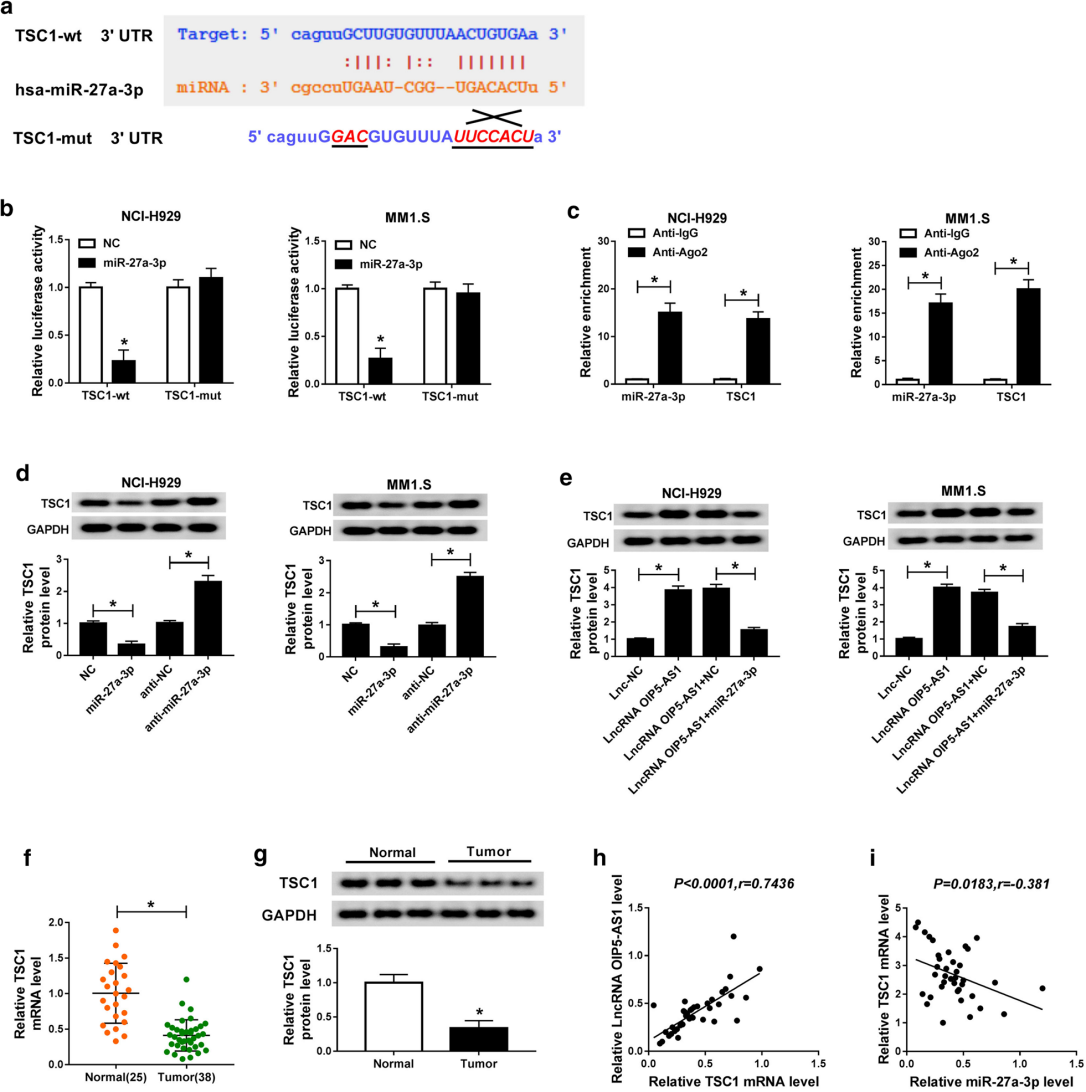
MiR-27a-3p could target TSC1 in MM. **a** The binding sites between miR-27a-3p and TSC1 3′UTR, as well as the mutant were exhibited. **b** Dual-luciferase reporter assay was carried out to measure the luciferase activity in TSC1-wt group and TSC1-mut group of NCI-H929 and MM1.S cells. **c** The levels of miR-27a-3p and TSC1 were examined after Ago2 or IgG RIP by qRT-PCR assay. **d** The expression of TSC1 protein in NCI-H929 and MM1.S cells transfected with NC, miR-27a-3p, anti-NC or anti-miR-27a-3p was tested by western blot assay. **e** The expression of TSC1 protein in NCI-H929 and MM1.S cells transfected with Lnc-NC, LncRNA OIP5-AS1, LncRNA OIP5-AS1 + NC or LncRNA OIP5-AS1 + miR-27a-3p was measured by western blot assay. **f**, **g** The mRNA and protein levels of TSC1 in bone marrows of 38 MM patients and 25 healthy donors were determined by qRT-PCR and western blot assays, respectively. **h, i** The correlation between the expression of TSC1 mRNA and OIP5-AS1, as well as between the expression of miR-27a-3p and TSC1 mRNA was determined via Pearson correlation analysis. ^*^P < 0.05

By feat of Starbase, we found that there were binding sites of miR-27a-3p on the 3′UTR of TSC1 (Fig. 5a). Dual-luciferase reporter assay demonstrated that introduction of miR-27a-3p prominently reduced the luciferase activity in TSC1-wt group of NCI-H929 and MM1.S cells, but it had no significant impact on the luciferase activity in TSC1-mut group (Fig. 5b). Additionally, RIP assay implied that miR-27a-3p could bind with TSC1 (Fig. 5c). As shown in Fig. 5d, miR-27a-3p lowered the protein level of TSC1 in NCI-H929 and MM1.S cells, while miR-27a-3p deficiency augmented expression of TSC1 protein. The protein level of TSC1 was elevated by OIP5-AS1 in NCI-H929 and MM1.S cells, but repressed via miR-27a-3p mimic (Fig. 5e). TSC1 expression was declined in bone marrows of MM patients relative to bone marrows of healthy donors, at mRNA and protein levels (Fig. 5f, g). And, a distinct positive correlation between the expression of TSC1 mRNA and OIP5-AS1, as well as an obvious inverse correlation between the expression of miR-27a-3p and TSC1 mRNA were observed in bone marrows of MM patients (Fig. 5h, i). Taken together, OIP5-AS1 upregulated TSC1 expression by serving as a sponge of miR-27a-3p.

### Silencing of TSC1 alleviated miR-27a-3p knockdown-mediated effects on the cellular behaviors of MM cells

**Fig. 6:**
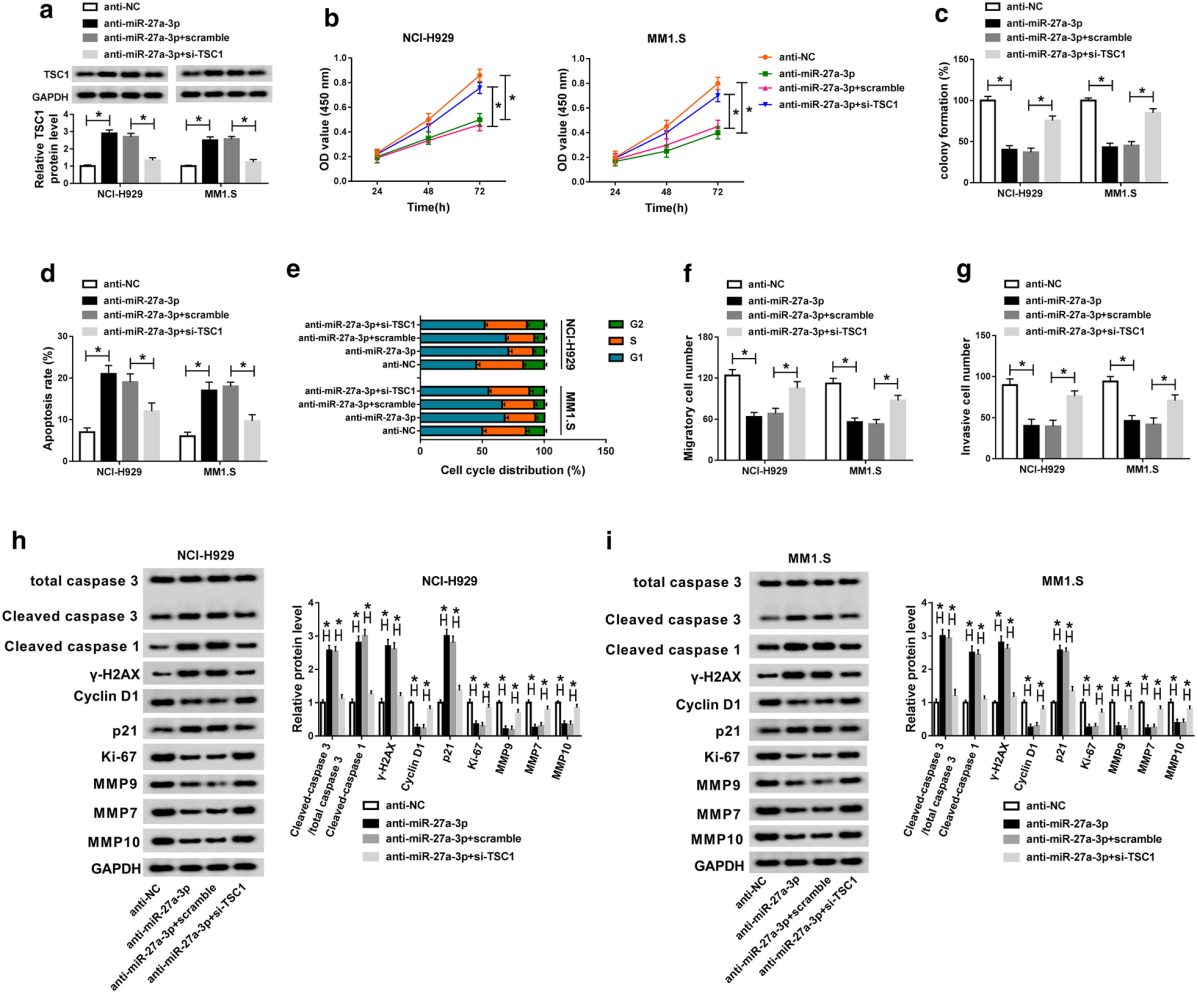
Silencing of TSC1 alleviated miR-27a-3p knockdown-mediated effects on the cellular behaviors of MM cells. NCI-H929 and MM1.S cells were transfected with anti-NC, anti-miR-27a-3p, anti-miR-27a-3p + scramble or anti-miR-27a-3p + si-TSC1. **a** The relative expression of TSC1 protein in transfected cells was detected by western blot assay. **b** Cell viability of transfected cells was examined via CCK-8 assay. **c** The colony formation ability of transfected cells was evaluated by colony formation assay. **d**, **e** The apoptosis rate and cell cycle distribution were detected through flow cytometry assay. **f**, **g** The migration and invasion abilities of transfected cells were measured via transwell assay. **h**, **i** The protein levels of Cleaved caspase 3/total caspase 3, Cleaved caspase 1, γ-H2AX, Cyclin D1, p21, Ki-67, MMP9, MMP7 and MMP10 were analyzed by western blot analysis. ^*^P < 0.05

To determine the role of miR-27a-3p in MM progression and the potential mechanism, NCI-H929 and MM1.S cells were transfected with anti-NC, anti-miR-27a-3p, anti-miR-27a-3p + scramble or anti-miR-27a-3p + si-TSC1. Silencing of TSC1 reversed the upregulation of TSC1 protein triggered by miR-27a-3p inhibitor (Fig. 6a). MiR-27a-3p knockdown conspicuously repressed the cell viability, colony formation ability, proliferative ability, migration and invasion capacities of NCI-H929 and MM1.S cells, which were all relieved by the additional si-TSC1 (Fig. 6b, c, Additional file 1: Fig. S1C, Fig. 6f, g). Silencing of TSC1 also vitiated miR-27a-3p inhibitor-induced cell apoptosis and cell cycle arrest at G1 phrase in NCI-H929 and MM1.S cells (Fig. 6d, e). As expected, miR-27a-3p inhibitor-mediated augment of Cleaved caspase 3/total caspase 3, Cleaved caspase 1, γ-H2AX and p21 levels, and the decline of Cyclin D1, Ki-67, MMP9, MMP7 and MMP10 levels in NCI-H929 and MM1.S cells were restored via TSC1 silencing (Fig. 6h, i). Altogether, miR-27a-3p knockdown inhibited MM evolvement by enhancing TSC1 expression.

### LncRNA OIP5-AS1 inhibited tumor growth in vivo

**Fig. 7:**
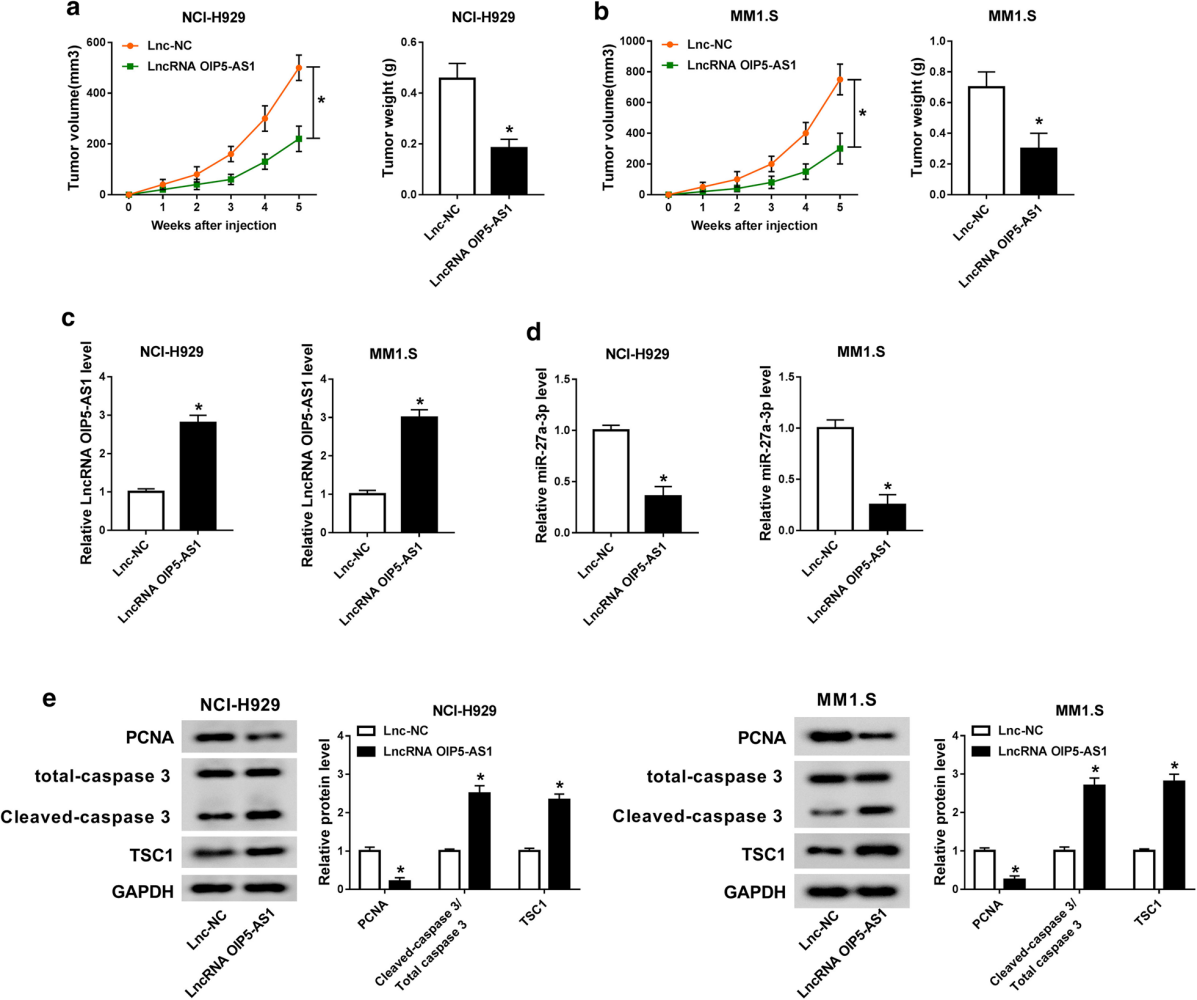
LncRNA OIP5-AS1 inhibited tumor growth in vivo. MM xenograft model was built by injecting NCI-H929 or MM1.S cells stably expressing LncRNA OIP5-AS1 or Lnc-NC into nude mice. **a**, **b** Tumor volume and weight were recorded. **c**, **d** Levels of OIP5-AS1 and miR-27a-3p were examined by qRT-PCR assay. **e** Protein levels of PCNA, Cleaved caspase 3/total caspase 3 and TSC1 were measured using western blot analysis. ^*^P < 0.05

We constructed MM xenograft model to explore the role of lncRNA OIP5-AS1 in vivo. NCI-H929 or MM1.S cells stably expressing LncRNA OIP5-AS1 or Lnc-NC were subcutaneously implanted into nude mice. As illustrated in Fig. 7a, b, the formed tumor in LncRNA OIP5-AS1 group displayed smaller volume and lower weight in contrast to Lnc-NC group. Furthermore, the expression level of OIP5-AS1 was elevated, while miR-27a-3p expression was declined in LncRNA OIP5-AS1 group with respect to Lnc-NC group (Fig. 7c, d). The protein level of PCNA was decreased, but levels of Cleaved caspase 3/total caspase 3 and TSC1 were promoted in LncRNA OIP5-AS1 group, which were uncovered by western blot analysis (Fig. 7e). In conclusion, lncRNA OIP5-AS1 could inhibit MM tumor growth in vivo.

## Discussion

It is widely accepted that multiple molecules and signaling pathways are implicated with the occurrence and development of MM, including lncRNAs [18]. In the current project, we proved that the suppressor role of lncRNA OIP5-AS1 in MM in vitro and in vivo. Besides, we firstly validated the regulation effect of OIP5-AS1/miR-27a-3p/TSC1 axis on MM progression.

Former works disclosed that lncRNA OIP5-AS1 was highly enriched in lung cancer [19], gastric cancer [20] and bladder cancer [21], performing as oncogene and predicting poor prognosis of cancer patients; and was downregulated in in basal-like tumors [22], cervical cancer [23] and MM [24], suggesting the dual functions of lncRNA OIP5-AS1 in human malignancies, which might be related to different tumor microenvironment. Here, we observed the downregulation of OIP5-AS1 in the bone marrows of MM patients. Consistent with the project of Yang et al. [11], we also confirmed the suppressor role of OIP5-AS1 in MM through a series of gain-of-function assays in vitro and in vivo. We inferred that OIP5-AS1 might be a treatment target for MM.

Mechanically, lncRNA could play as sponge of miRNA to regulate miRNA expression, further modulate tumorigenesis of human cancers [25, 26]. For instance, lncRNA OIP5-AS1 aggravated oral squamous cell carcinoma progression by sponging miR-338-3p to upregulate NRP1 [27]. Besides, OIP5-AS1 accelerated CDK14 abundance to contribute to osteosarcoma tumorigenesis via functioning as a sponge of miR-223 [28]. Here, we first validated that OIP5-AS1 could sponge miR-27a-3p by bioinformatics analysis, dual-luciferase reporter and RNA immunoprecipitation assays. Apart from this, OIP5-AS1-mediated inhibitory impact on the tumorigenic properties was weakened by introduction of miR-27a-3p. Therefore, we concluded that OIP5-AS1 caused MM development suppression by sponging miR-27a-3p.

MiR-27a-3p was identified to be a tumor oncogene in osteosarcoma [29], gastric cancer [30] and MM [16]. Likewise, from our data, miR-27a-3p was highly enriched in the bone marrows of MM patients. Functional analyses revealed that loss of miR-27a-3p could hamper proliferation and metastasis of MM NCI-H929 and MM1.S cells. It is worth mentioning that miR-27a-3p exerts its biological roles by lowering the expression levels of its target mRNAs, like inhibitor of growth family member 5 (ING5), B cell translocation gene 2 (BTG2) and SPRY2 [16, 29, 30].

In this project, we confirmed that TSC1 was a function target of miR-27a-3p. TSC1 could play significant part in multiple myeloma, which was suggested by Du et al. [17]. Additionally, TSC1 was validated to be downregulated in MM samples, and abolished miR-19b-induced MM development [31]. The tumor suppressor role of TSC1 was also corroborated in prostate cancer and high-grade serous ovarian carcinoma [32, 33]. Consistently, we found that TSC1 enrichment was apparently declined in MM as well. And TSC1 interference abrogated the effects of miR-27a-3p knockdown on the cellular behaviors of MM NCI-H929 and MM1.S cells. What’s more, lncRNA OIP5-AS1 activated TSC1 expression by sponging miR-27a-3p. As a consequence, OIP5-AS1 reduced MM progression by regulating miR-27a-3p/TSC1 axis.

There still exist some deficiencies in our investigation. Signal pathways associated with the evolvement of MM, like NF-κB pathway [9], PI3K/AKT pathway [34] and TGF-β/Smad pathway [35], would to be investigated to figure out whether they were involved in the lncRNA OIP5-AS1-mediated or miR-27a-3p-mediated MM progression.

## Conclusion

In conclusion, lncRNA OIP5-AS1 could repress MM progression in vitro and in vivo, probably through the modulation of miR-27a-3p/TSC1 axis at least. This study might supply a novel mechanism for confirming the potential of lncRNA OIP5-AS1 to be a therapeutic agent of multiple myeloma.

## Supplementary information

**Additional file 1: Fig. S1.** BrdU incorporation in transfected MM cells. (A) BrdU staining assay for BrdU incorporation in NCI-H929 and MM1.S cells transfected with LncRNA OIP5-AS1 or Lnc-NC (A), transfected with Lnc-NC, LncRNA OIP5-AS1, LncRNA OIP5-AS1 + NC or LncRNA OIP5-AS1 + miR-27a-3p (B), transfected with anti-NC, anti-miR-27a-3p, anti-miR-27a-3p + scramble or anti-miR-27a-3p + si-TSC1 (C). *P < 0.05.

**Additional file 2: Fig. S2.** Overexpression of OIP5-AS1 hampered proliferation and metastasis, but facilitated apoptosis of MM RPMI-8226 cells. MM RPMI-8226 cells were transfected with LncRNA OIP5-AS1 or Lnc-NC. (A) The relative expression of lncRNA OIP5-AS1 in transfected cells was analyzed by qRT-PCR assay. (B) Cell viability of transfected cells was analyzed by CCK-8 assay. (C) BrdU incorporation in transfected cells was determined by BrdU staining assay. (D) The colony formation ability of transfected cells was monitored via colony formation assay. (E–F) The apoptosis rate and cell cycle distribution were tested through flow cytometry assay. (G-H) The migration and invasion abilities of transfected cells were examined by transwell assay. (I) The protein levels of Cleaved caspase 3/total caspase 3, Cleaved caspase 1, γ-H2AX, Cyclin D1, p21, Ki-67, MMP9, MMP7 and MMP10 were evaluated by western blot analysis. *P < 0.05.

## Data Availability

The datasets used and/or analyzed during the current study are available from the corresponding author on reasonable request.

## Abbreviations

MM: Multiple myeloma
TSC1: Tuberous sclerosis 1
