# Supplementary figures and images for: Long non-coding RNA OIP5-AS1 suppresses multiple myeloma progression by sponging miR-27a-3p to activate TSC1 expression
Source: Cancer Cell Int. 2020 May 7;20:155. doi: 10.1186/s12935-020-01234-7 (PMC7206794; doi:10.1186/s12935-020-01234-7)

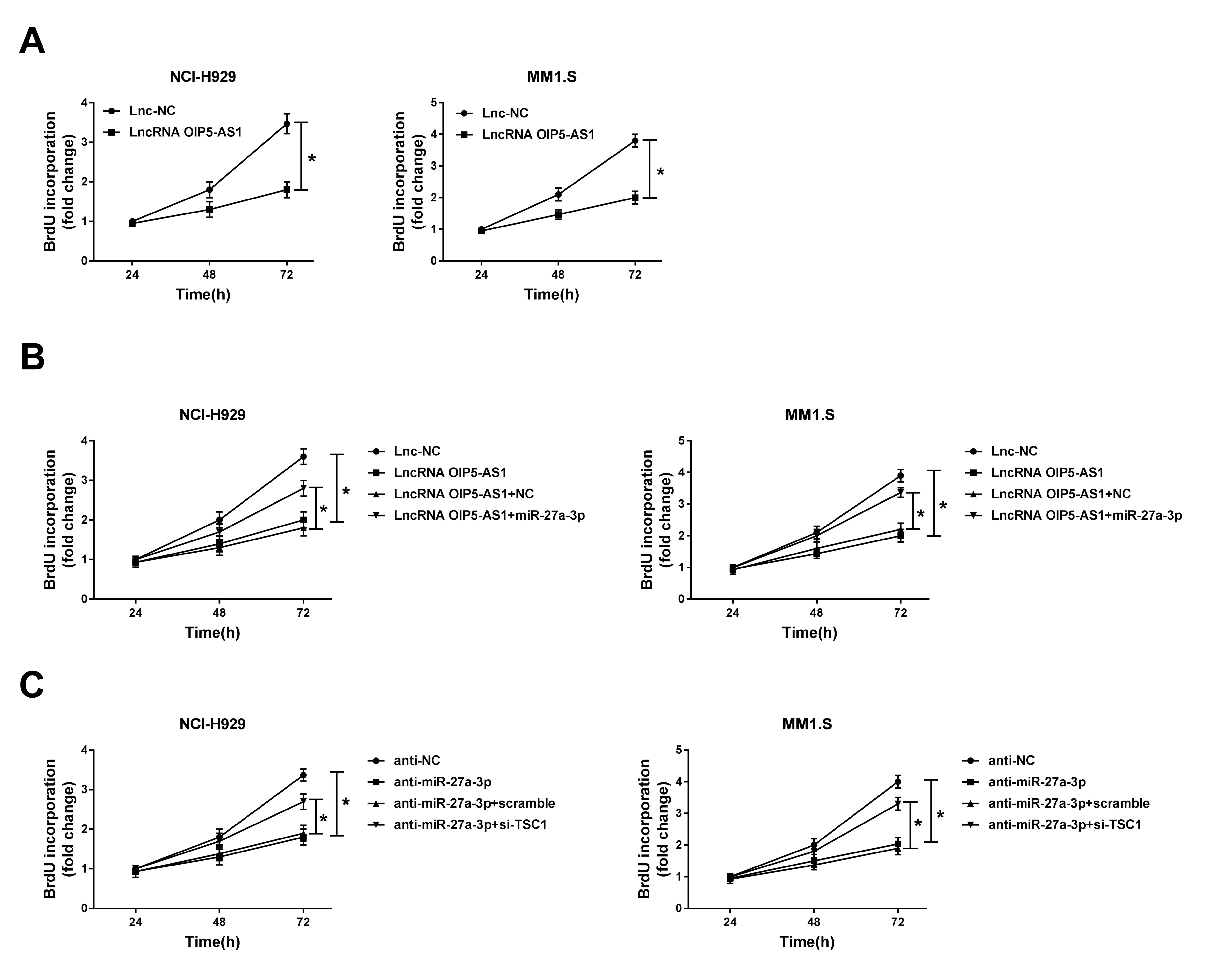

Supplement: Supplementary file 1 — Additional file 1: Fig. S1. BrdU incorporation in transfected MM cells. (A) BrdU staining assay for BrdU incorporation in NCI-H929 and MM1.S cells transfected with LncRNA OIP5-AS1 or Lnc-NC (A), transfected with Lnc-NC, LncRNA OIP5-AS1, LncRNA OIP5-AS1 + NC or LncRNA OIP5-AS1 + miR-27a-3p (B), transfected with anti-NC, anti-miR-27a-3p, anti-miR-27a-3p + scramble or anti-miR-27a-3p + si-TSC1 (C). *P < 0.05. [file 12935_2020_1234_MOESM1_ESM.jpg]

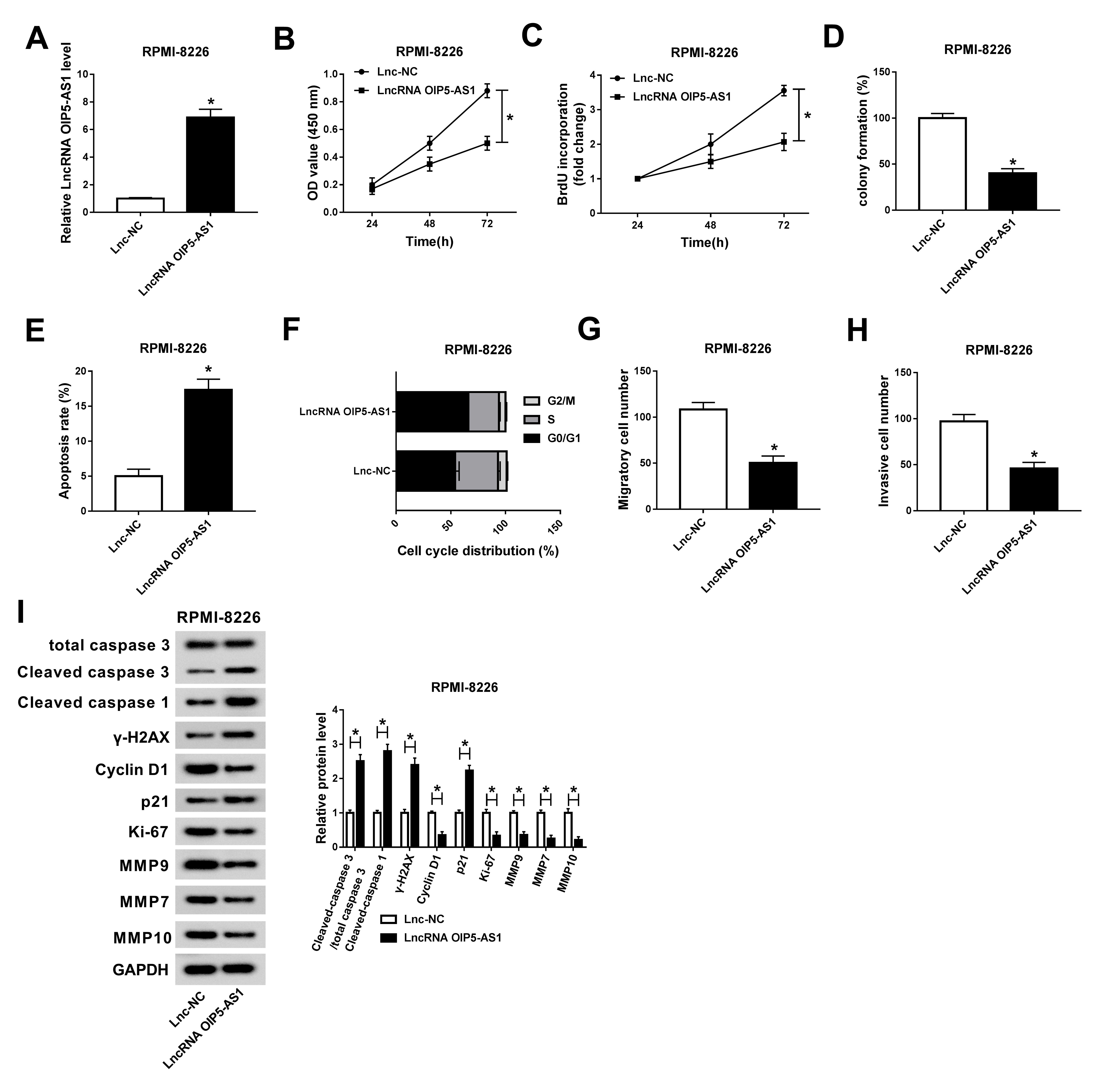

Supplement: Supplementary file 2 — Additional file 2: Fig. S2. Overexpression of OIP5-AS1 hampered proliferation and metastasis, but facilitated apoptosis of MM RPMI-8226 cells. MM RPMI-8226 cells were transfected with LncRNA OIP5-AS1 or Lnc-NC. (A) The relative expression of lncRNA OIP5-AS1 in transfected cells was analyzed by qRT-PCR assay. (B) Cell viability of transfected cells was analyzed by CCK-8 assay. (C) BrdU incorporation in transfected cells was determined by BrdU staining assay. (D) The colony formation ability of transfected cells was monitored via colony formation assay. (E–F) The apoptosis rate and cell cycle distribution were tested through flow cytometry assay. (G-H) The migration and invasion abilities of transfected cells were examined by transwell assay. (I) The protein levels of Cleaved caspase 3/total caspase 3, Cleaved caspase 1, γ-H2AX, Cyclin D1, p21, Ki-67, MMP9, MMP7 and MMP10 were evaluated by western blot analysis. *P < 0.05. [file 12935_2020_1234_MOESM2_ESM.jpg]
